# A Rare Presentation of Genitourinary Melioidosis: Isolation of Burkholderia pseudomallei From a Prostatic Abscess in a Patient From Puducherry

**DOI:** 10.7759/cureus.90443

**Published:** 2025-08-18

**Authors:** Disha Gautam, Rushika Saksena, C Meghna

**Affiliations:** 1 Clinical Microbiology, Vardhman Mahavir Medical College and Safdarjung Hospital, New Delhi, IND; 2 Microbiology, Pondicherry Institute of Medical Sciences, Puducherry, IND

**Keywords:** burkholderia pseudomallei, farmer, genitourinary infection, melioidosis, prostate abscess

## Abstract

Melioidosis, caused by the facultative intracellular Gram-negative bacterium *Burkholderia pseudomallei*, presents with diverse clinical manifestations ranging from asymptomatic carriage to life-threatening infections. We report a rare case of genitourinary melioidosis presenting as a prostatic abscess in a 70-year-old male patient with chronic kidney disease from southern India. The patient presented with fever, breathlessness, and scrotal swelling. Imaging revealed multiple prostatic abscesses with urine cultures growing *B. pseudomallei*, confirmed by biochemical tests and automated identification systems. The patient was empirically treated with IV clindamycin (600 mg q8h) and IV piperacillin-tazobactam (4.5 gm q8h) along with inotropes and fluids. Timely confirmation of genitourinary melioidosis led to escalation of antibiotics to IV ceftazidime 2 gm q6h for four weeks, followed by oral trimethoprim-sulfamethoxazole for three months, leading to complete clinical and microbiological recovery. This case highlights the importance of early recognition and accurate microbiological diagnosis of *B. pseudomallei* in atypical presentations to ensure timely and effective therapy, especially in endemic regions and in patients with risk factors such as chronic kidney disease.

## Introduction

Melioidosis is an infectious disease caused by *Burkholderia pseudomallei (B. pseudomallei)*, a facultative intracellular, Gram-negative bacillus found in soil and surface water. The disease is endemic in Southeast Asia and northern Australia, with increasing recognition in South Asia, China, and parts of Central and South America. Sporadic cases have also been reported in non-endemic regions due to travel or imported exposure. The clinical presentation of melioidosis is highly variable, ranging from asymptomatic colonization to rapidly progressive sepsis. It most commonly affects individuals with underlying risk factors such as diabetes mellitus, chronic alcoholism, chronic kidney disease, chronic pulmonary disease, immunosuppression, and malignancy [1,2]. Involvement of the genitourinary tract is uncommon, and prostatic abscesses due to *B. pseudomallei* are considered rare but increasingly recognized, particularly in endemic areas. Several case reports have documented such presentations, emphasizing the diagnostic challenge and the need for a high degree of clinical suspicion.

We report a rare case of *B. pseudomallei* isolated from the urine of a patient diagnosed with a prostatic abscess. This case underscores the critical importance of early recognition, appropriate microbiological identification, and timely management of this potentially fatal organism, particularly in regions where melioidosis is endemic or in patients with a relevant history of exposure.

## Case presentation

A 70-year-old male patient, a farmer by occupation, with chronic kidney disease (CKD), and a resident of Puducherry from southern India, came to the emergency department with complaints of fever, breathlessness, and decreased food intake for three days. The patient was febrile, tachypneic, with a blood pressure of 110/68 mmHg. On local examination, a tender, fluctuant swelling of 2 × 3 cm was observed on the upper part of the scrotum (suggestive of a localized abscess). A pulsatile swelling around the umbilicus was also noted (suggestive of an aneurysm). The patient was investigated, and his complete blood count (CBC) showed neutrophilic predominance. The other relevant tests are also shown below (Table 1).

**Table 1:** Laboratory parameters of the patient with their reference values

Laboratory Investigations	Result	Reference values
White blood cells (WBC)	10,090/µL	5000-10,000/µl
Hemoglobin	6.7 g/dL	13.3-16.2 g/dL
Erythrocyte sedimentation rate (ESR)	88 mm/h	<20 mm/h
C-reactive protein (CRP)	86 mg/L	0-3 mg/L
Urea	84 mg/dL	13-43 mg/dL
Creatinine	2.6 mg/dL	0.60-1.30 mg/dL

**Figure 1:**
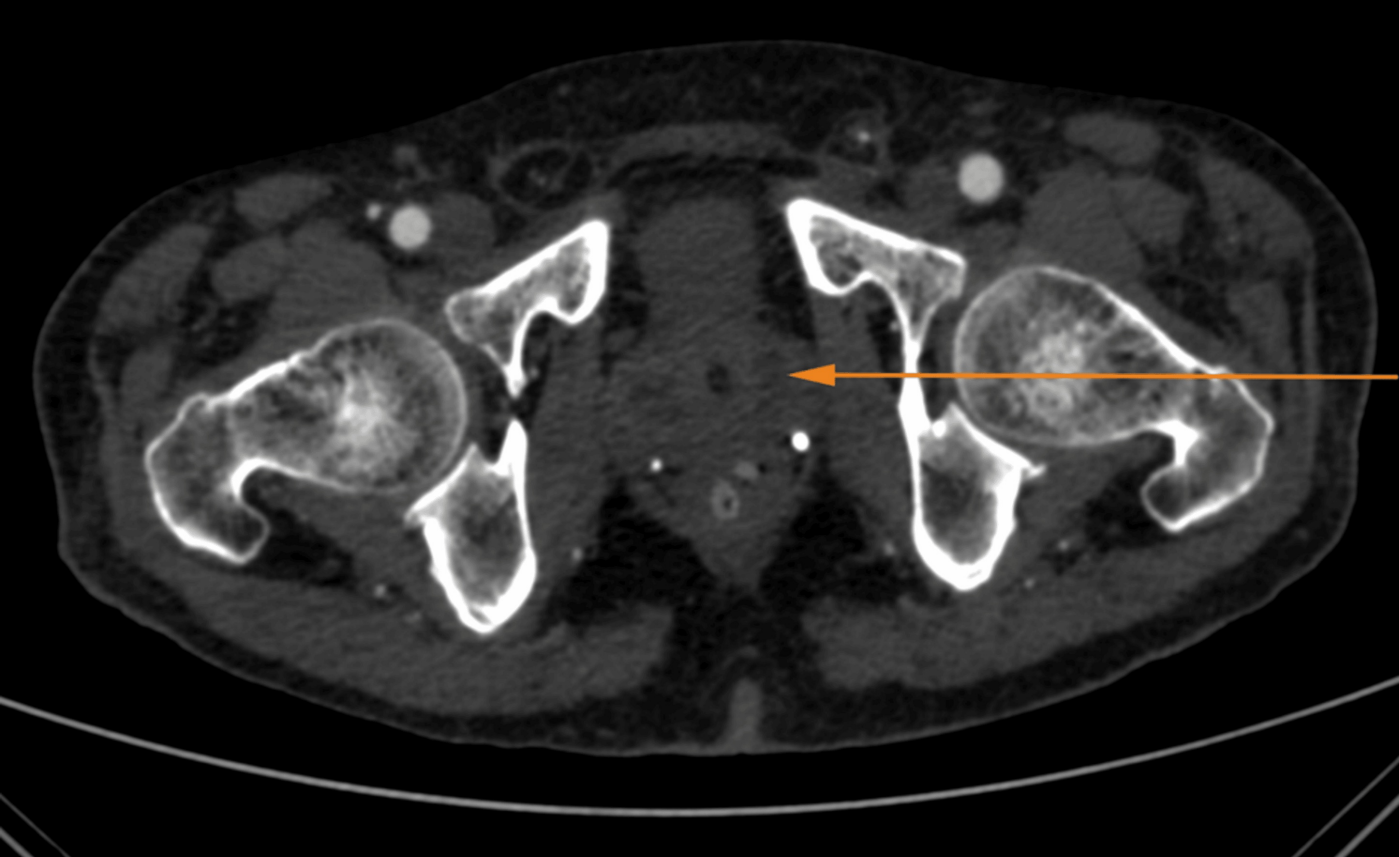
The computed tomography (CT) abdominal angiography showing the enlarged prostate with peripheral enhancing hypodense lesion (arrow), indicating a prostatic abscess

**Figure 2:**
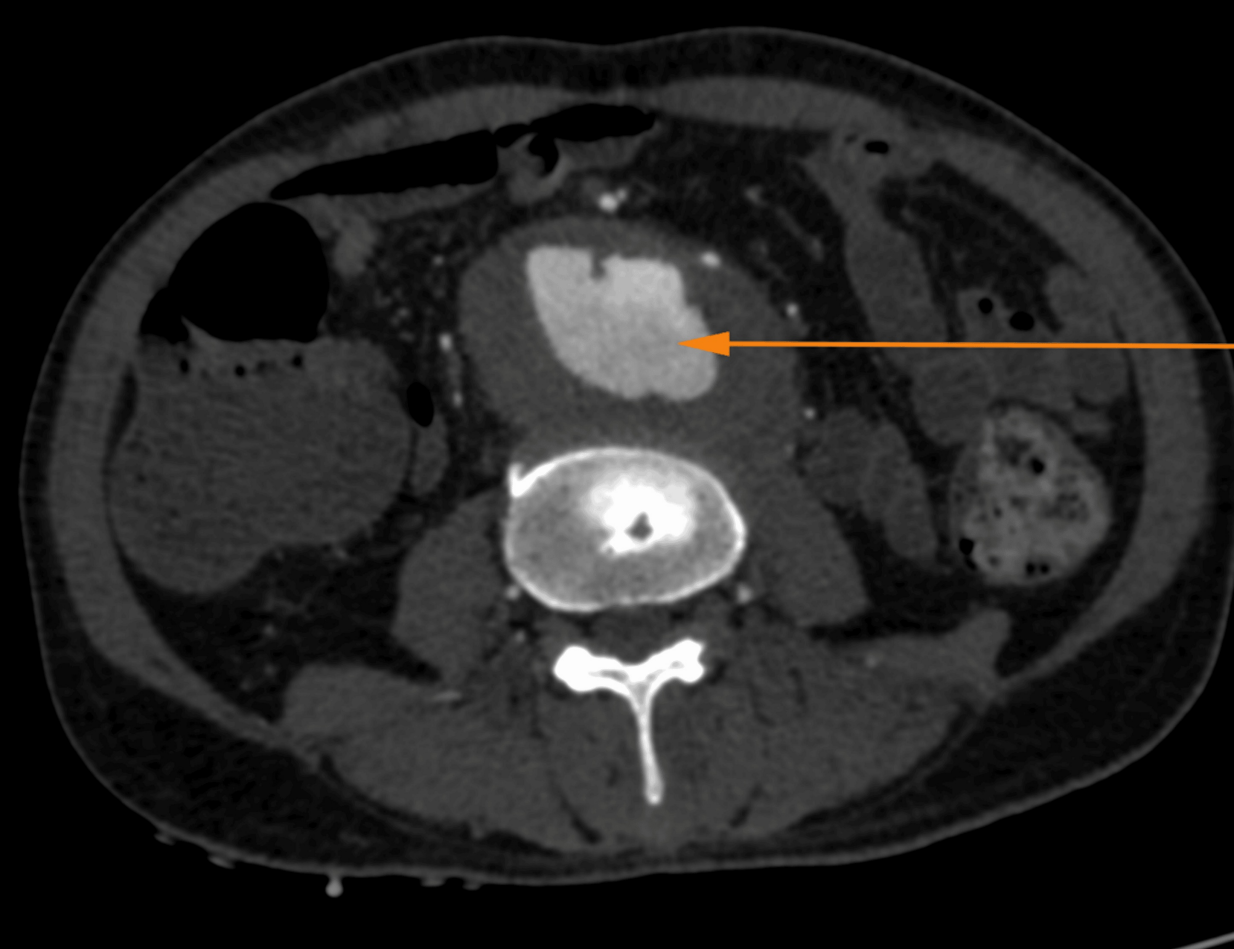
CT showing sacular contrast filled outpouching (arrow) in the posterolateral aspect of the aorta, suggestive of an aneurysm

The computed tomography (CT) abdominal angiography revealed an enlarged prostate (~34 cc) and seminal vesicle with multiple irregular peripherally enhancing hypodense lesions, likely to be abscesses (the largest measuring ~2.3 X 1.5 cm) and sacular contrast filled outpouching in the postero-lateral aspect of the aorta, likely to be an abdominal aortic aneurysm (Figures 1, 2).

**Figure 3:**
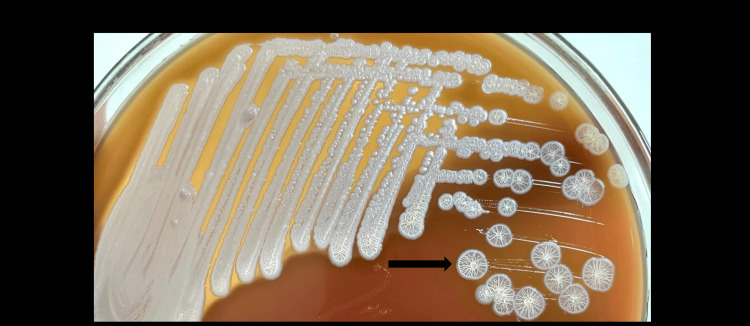
Colony morphology of approximately 2-3 mm in size, white colored, round, smooth, elevated, and dry wrinkled colonies on sheep blood agar after 48 hours of incubation

**Figure 4:**
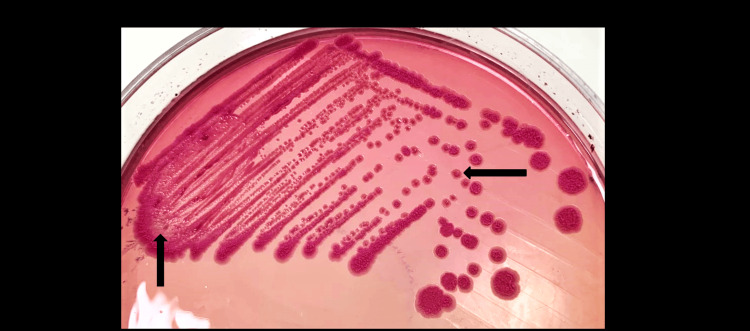
Non-lactose fermenting colonies (2-3 mm), turning dark pink, dry, and wrinkled with radiating ridges on MacConkey agar after 48-72 hours of incubation

In view of the multiple prostatic abscesses, blood and urine cultures were advised to rule out sepsis. As no specific pathogen was suspected, urine specimen was inoculated on 5% sheep blood agar and MacConkey agar (MAC) and incubated aerobically at 37°C. After 24 hrs of incubation, growth of small, pinpoint colonies (colony count >10^5^ CFU/mL) was observed on both media. On further incubation, characteristic colonies of 2 to 3 mm in size, smooth non-hemolytic with a metallic sheen were observed (Figures 3, 4).

**Figure 5:**
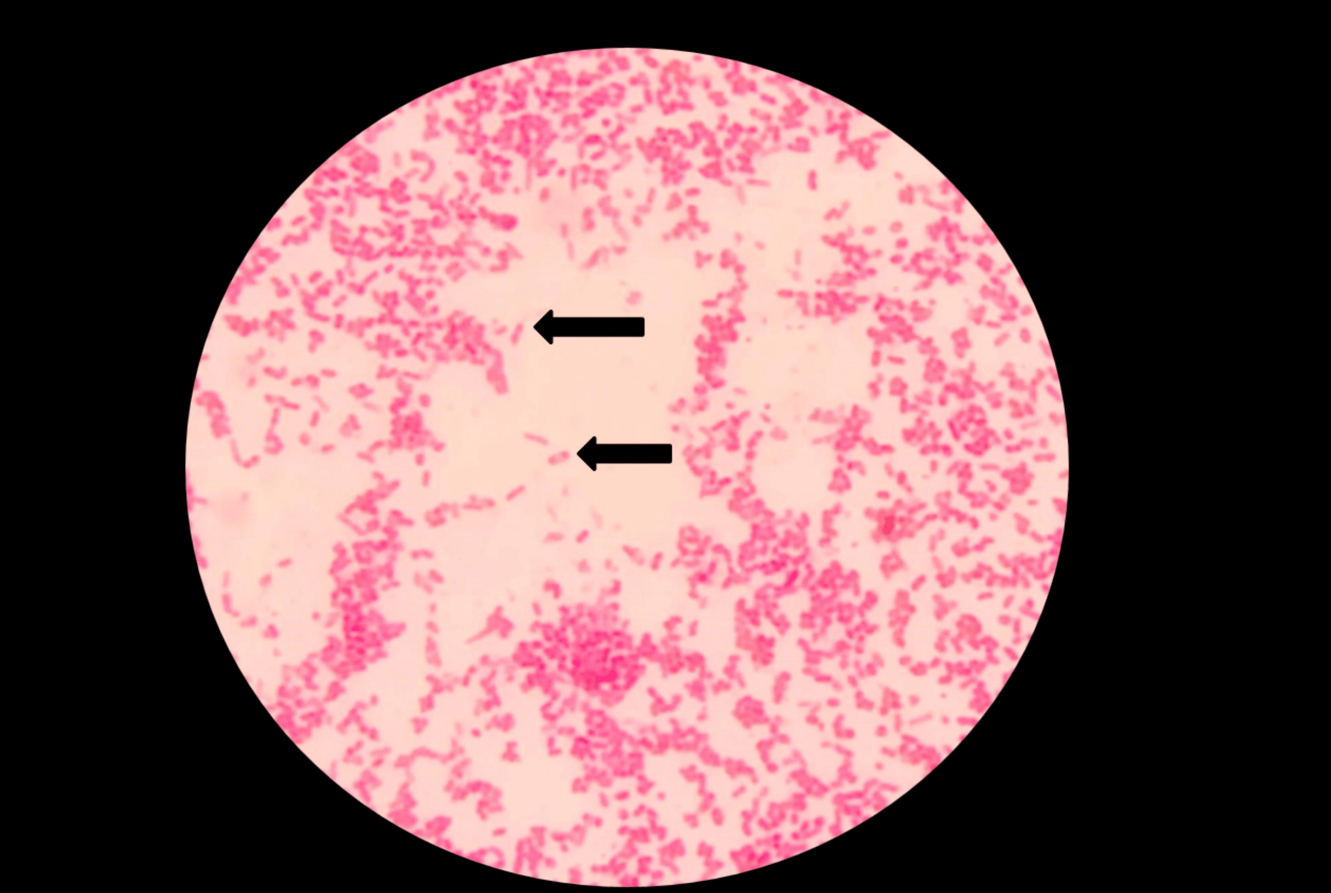
Gram staining showing gram negative bacilli with safety pin appearance (100x Oil Immersion Field)

The organism was non-lactose fermenting, oxidase and catalase positive, and Gram-negative bacilli with bipolar staining (safety pin appearance) on Gram staining (Figure 5).

The isolate was presumptively identified as *B. pseudomallei* on conventional biochemical tests and triple disk test (resistant to gentamicin (10 µg) and polymyxin B (10 µg), and sensitive to amoxicillin-clavulanate (20/10 µg)) [3]. On day three, identification and antimicrobial susceptibility testing were performed by VITEK-2 Compact (bioMerieux, Marcy l’Etoile, France). Identification was confirmed as *B. pseudomallei* (99% confidence) and the isolate was susceptible to amoxicillin-clavulanic acid, ceftazidime, meropenem and trimethoprim-sulphamethoxazole (TMP-SMX) (interpreted using Clinical and Laboratory Standard Institute (CLSI) guidelines M45, 2019) [4]. The blood culture was reported sterile after seven days of incubation.

Based on these findings, the patient was diagnosed as a case of genitourinary melioidosis. On day three of admission, intensive phase therapy with IV ceftazidime 2 gm q6h was initiated for four weeks. The patient was treated with two units of packed cell volume (PCV) in view of the low hemoglobin count and antiplatelets (to prevent thrombosis due to aneurysm). The patient was discharged on oral TMP-SMX with dosage of 160 mg TMP/800 mg SMX, two tablets twice daily for three months. After three months of the eradication phase, the patient showed clinical and microbiological recovery.

## Discussion

Genitourinary disease is a relatively rare presentation of melioidosis. In a study from India, over the span of ten years, only 20 patients with genitourinary melioidosis were identified, all of whom were male patients [5]. Most patients (90%) had chronic disease (duration of more than two months), and 55 % also had bacteremia. The prostate was the most commonly involved organ, accounting for 50% of cases. The kidney was the second most common site, presenting as pyelonephritis and renal abscesses [5]. Other sporadic cases of genitourinary melioidosis reported in recent times show that diabetes mellitus and urinary tract abnormalities (mainly lithiasis and ureteral obstruction) are the predisposing risk factors for this infection in adults [6-8].

The treatment of melioidosis consists of two phases: an intensive phase and an eradication phase. The antibiotics recommended in the intensive phase are intravenous meropenem or ceftazidime [9,10]. For deep abscesses, IV meropenem or ceftazidime is recommended in the intensive phase for four weeks, followed by oral TMP-SMX or amoxicillin-clavulanic acid in the eradication phase for 12 weeks. As our patient had multiple prostatic abscesses, he was treated for deep-seated infection in accordance with Darwin guidelines after the laboratory-confirmed diagnosis of genitourinary melioidosis [10].

As genitourinary infections with* B. pseudomallei* are rare, several diagnostic challenges impede timely and accurate detection of these infections. A low index of suspicion amongst clinicians means that many patients are not being tested for melioidosis at all. Even when cultures are requested, often these Gram-negative non-fermenters from urine cultures are misidentified as *Pseudomonas spp.* either due to a lack of awareness or unavailability of automated identification systems [11].

This case is therefore presented to highlight a rare yet severe manifestation of melioidosis in the form of a deep-seated genitourinary abscess, occurring in an endemic region like India. The prompt diagnosis and timely initiation of appropriate antimicrobial therapy prevented complications such as bacteremia or disseminated infection, resulting in a favorable clinical outcome.

## Conclusions

Genitourinary melioidosis accounts for a small proportion of all cases of melioidosis; prostate and kidney involvement are uncommon, often accompanied by spread to other sites. Early suspicion and definitive laboratory confirmation-via culture or newer molecular methods-are essential for effective management. In conclusion, clinicians should consider melioidosis as a rare but important differential diagnosis in patients presenting with multiple abscesses in the lower genitourinary tract, particularly from endemic regions such as southern India. This uncommon case underscores the need to maintain a high index of suspicion for melioidosis in such presentations. Increased awareness among clinicians and microbiologists plays a crucial role in the timely diagnosis and appropriate treatment of this under-reported disease.
